# Different composition of intraocular immune mediators in Posner-Schlossman-Syndrome and Fuchs’ Uveitis

**DOI:** 10.1371/journal.pone.0199301

**Published:** 2018-06-26

**Authors:** Dominika Pohlmann, Stephan Schlickeiser, Sylvia Metzner, Matthias Lenglinger, Sibylle Winterhalter, Uwe Pleyer

**Affiliations:** 1 Department of Ophthalmology, Charité – Universitätsmedizin Berlin, corporate member of Freie Universität Berlin, Humboldt-Universität zu Berlin, and Berlin Institute of Health, Berlin, Germany; 2 Institute of Medical Immunology, Charité – Universitätsmedizin Berlin, Berlin, Germany; Oregon Health and Science University, UNITED STATES

## Abstract

Posner-Schlossman-Syndrome (PSS) is clinically characterized by acute, recurrent, mild, unilateral uveitis anterior accompanied by elevated intraocular pressure (IOP). Fuchs´ Uveitis (FU) is a chronic, low-grade-inflammatory disorder, involving anterior uvea and vitreous. The clinical findings show remarkable similarities as well as differences. In our study, we determine the composition of immune mediators in aqueous humor of patients with PSS and FU and evaluate if immune mediators play a crucial role in specific viral intraocular inflammation and IOP rises. Aqueous humor samples from 81 uveitis patients (= eyes) presenting with either PSS or FU were collected at one time point. Local intraocular antibody synthesis to rubella virus was confirmed in 65 patients, whereas 16 were tested positively for human cytomegalovirus. Thirteen patients with PSS and 10 patients with FU were treated with glaucoma medications. Additionally, 11 cataract patients acted as control group. Immune mediator concentrations were measured by Bio-Plex Pro assay. We observed in both PSS (IFN-γ: 174.9 pg/mL; TNF-α: 25.1 pg/mL) and FU (IFN-γ: 25.4 pg/mL; TNF-α: 27.2 pg/mL) groups a significantly increased level of T-helper 1 immune mediators compared to controls (IFN-γ, TNF-α: 0 pg/mL) [median]. Notably, PSS patients (IL-1RA: 73.4 pg/mL; IL-8: 199.4 pg/mL; IL-10: 33.4 pg/mL; IP-10: 126350 pg/mL) showed a stronger and more active ocular inflammatory response, than FU patients (IL-1RA: 4.3 pg/mL; IL-8: 72.4 pg/mL; IL-10: 1.6 pg/mL; IP-10: 57400 pg/mL). Furthermore, a negative correlation between mediators and IOP was seen in the PSS group, potentially caused by acetazolamide-treatment. Our findings show that immune mediators play a crucial role in specific viral intraocular inflammation and influence IOP levels. Remarkable similarities but also significant differences of immune mediator concentrations are apparent in PSS compared to FU. High concentrations of IL-1RA, IL-8, IL-10, and IP-10 correlate with active inflammation in PSS, while FU may trigger chronic inflammation. Our data also substantiated a very similar composition of cytokines in those patients from the PSS group suffering from ocular hypertension and thus offers a potential explanation model for a negative correlation between mediators and IOP.

## Introduction

Posner-Schlossman-Syndrome (PSS) typically presents with acute, recurrent, unilateral attacks of markedly raised intraocular pressure (IOP) associated with the presence of cells in the anterior chamber and sparse keratic precipitates. Iris atrophy is also frequently reported, whereas posterior synechiae typically are not [1]. Fuchs’ uveitis (FU) is characterized by similar findings, including a mild unilateral inflammation of the anterior chamber with stellate keratic precipitates. It follows the course of a chronic, low-grade anterior uveitis. Heterochromia and iris atrophy may be present, and early cataract formation is common [2]. Because of these similar clinical features, PSS may be inadvertently misdiagnosed as FU.

Chee et al. showed that up to 52% of PSS patients were tested positively for cytomegalovirus (CMV) DNA. It is interesting to note that in their study, also 42% of presumed FU patients were CMV positive for viral DNA [3]. However, no other virus in aqueous humor was tested in this study. In 2004, Quentin and Reiber reported that FU is closely related to rubella virus (RV) antibodies in aqueous humor [4]. This was confirmed in subsequent studies that consistently demonstrated increased intraocular antibody synthesis against RV in clinical FU patients with remarkable 100% sensitivity and 100% specificity [5–8].

Clinically, PSS and FU display various similarities but also subtle differences, presumably attributable to the response of the eye. Therefore, we examined patients with clinical characteristics of PSS and FU. To confirm the underlying etiology, we analyzed CMV- and RV-specific immunoglobulin G (IgG) using aqueous humor and serum samples. Furthermore, we measured immune mediators in aqueous humor of PSS patients with positive CMV, and FU patients with positive RV-antibody synthesis, and then compared results across groups. Our hypothesis is that immune mediators play a crucial role in the context of specific viral intraocular inflammation and intraocular pressure (IOP) levels.

## Methods

### Inclusion of patients

This study was performed in accordance with the standards of the Declaration of Helsinki and approved by the local ethics committee (EA4/054/16) of Charité University Medicine Berlin. Written informed consent was obtained from each participating patient.

From January 2009 to May 2014, a total of 81 patients with PSS (16) and FU (65) were registered at the Department of Ophthalmology, Charité University Medicine Berlin. Aqueous humor samples were obtained for routine diagnostic purposes from patients with classical clinical signs of PSS or FU while the disease was active. The intraocular antibody synthesis to RV and CMV was confirmed by using the antibody index (AI) described in literature [9–15]. Inclusion criteria for PSS and FU patients were, beside of positive AI (CMV = PSS; RV = FU), the presence of a) low grade anterior uveitis, b) fine or focal, stellate keratic precipitates, c) absence of posterior synechiae, d) IOP>21 mmHg, e) heterochromia and/or iris stromal atrophy, f) early cataract formation or already pseudophakic eyes. Four out of these six principal signs had to be present for inclusion in the study. Additionally, anterior vitreous involvement had to be evident in FU patients [9]. Inflammation was evaluated using the scoring criteria set out by the Standardization of Uveitis Nomenclature (SUN) working group. The anatomic location was recorded additionally to disease onset, duration, course and activity [16]. A group of patients undergoing elective cataract surgery served as controls.

### Sample collection and processing

Aqueous humor and blood samples were collected simultaneously. Patients were given topical anesthesia, and a 31-gauge insulin syringe was inserted at the peripheral cornea parallel to the iris. Under direct microscopic view, approximately 100 μL of aqueous humor was extracted. For cataract controls, aqueous humor aspiration was performed at the beginning of surgery. Samples were cryopreserved at -80°C until further analysis, then defrosted and centrifuged at 150 g for 1 minute at room temperature. Twenty-one samples were diluted to obtain the defined volume of 50μL.

### Antibody index (AI)

A modified ELISA technique (Enzynost ^**®**^, Dade Behring Marburg, Germany) was used to detect antibodies in aqueous humor and serum, diluted to an IgG level of 1 mg/dL after total IgG in the serum and aqueous humor were measured [9,15]. A comparison of photometric signals of ΔE > 0.2 allowed for detection of intraocular IgG antibodies to CMV, RV, herpes simplex virus (HSV), and varicella zoster virus (VZV). The antibody index (AI) was determined using the Goldmann-Witmer-Desmonts coefficient [12,15]. Diagnosis of PSS was confirmed by AI > 3.0, ΔE > 0.200 for CMV, and of FU by AI > 3.0, ΔE > 0.200 for RV. HSV and VZV antibodies were also examined in all patients. All patients of the control group had been tested negative for either RV, CMV, VZV, and HSV.

### Immune mediator analysis

Bio-Plex ProTM magnetic colour-bead-based multiplex assay (Bio-Rad Laboratories, Inc. Hercules, CA) was used to measure the concentration of a total of 27 immune mediators: Eotaxin, Fibroblast growth factor basic (FGFbasic), Granulocyte-colony stimulating factor (G-CSF), Granulocyte macrophage colony-stimulating factor (GM-CSF), Interleukin-1 receptor antagonist (IL-1RA), IL-1b, IL-2, IL-4, IL-5, IL-6, IL-7, IL-8, IL-9, IL-10, IL-12, IL-13, IL-15, IL-17, Interferon-gamma (IFN-γ), Interferon gamma-induced protein 10 (IP-10), Macrophage inflammatory proteins 1 alpha and beta (MIP-1α and MIP-1β), Monocyte chemotactic protein 1 (MCP1), Platelet-derived growth factor (PDGF), Regulated upon Activation Normal T cell Expressed and Secreted (RANTES), Tumor-Necrosis-Factor-alpha (TNF-α), and Vascular Endothelial Growth Factor (VEGF). The assay was conducted according to manufacturer’s instruction. Fifty microliters of aqueous humor samples were used. Data analysis was performed by Bio-Plex Manager TM software 1.1.

### Statistical analysis

Data were analyzed using GraphPad Prism 6 (GraphPad Software, La Jolla, CA) and R version 3.3 (34). For differences in cytokine concentrations, non-parametric Mann-Whitney testing was performed. Two-tailed, non-parametric Spearman method was applied to assess the correlation between variables. A p-value of p ≤ 0.05 was defined as significant. Due to the exploratory nature of this study, p-values were not corrected for multiple testing. R was used for generating a heatmap representation of data, where Pearson correlation matrices of raw cytokine concentrations or of mean-centered and sigma-normalized data served as input for hierarchical clustering (using Ward’s minimum variance method) of cytokines (heatmap row dendrogram) or patient groups (column dendrogram), respectively.

## Results

**Fig 1:**
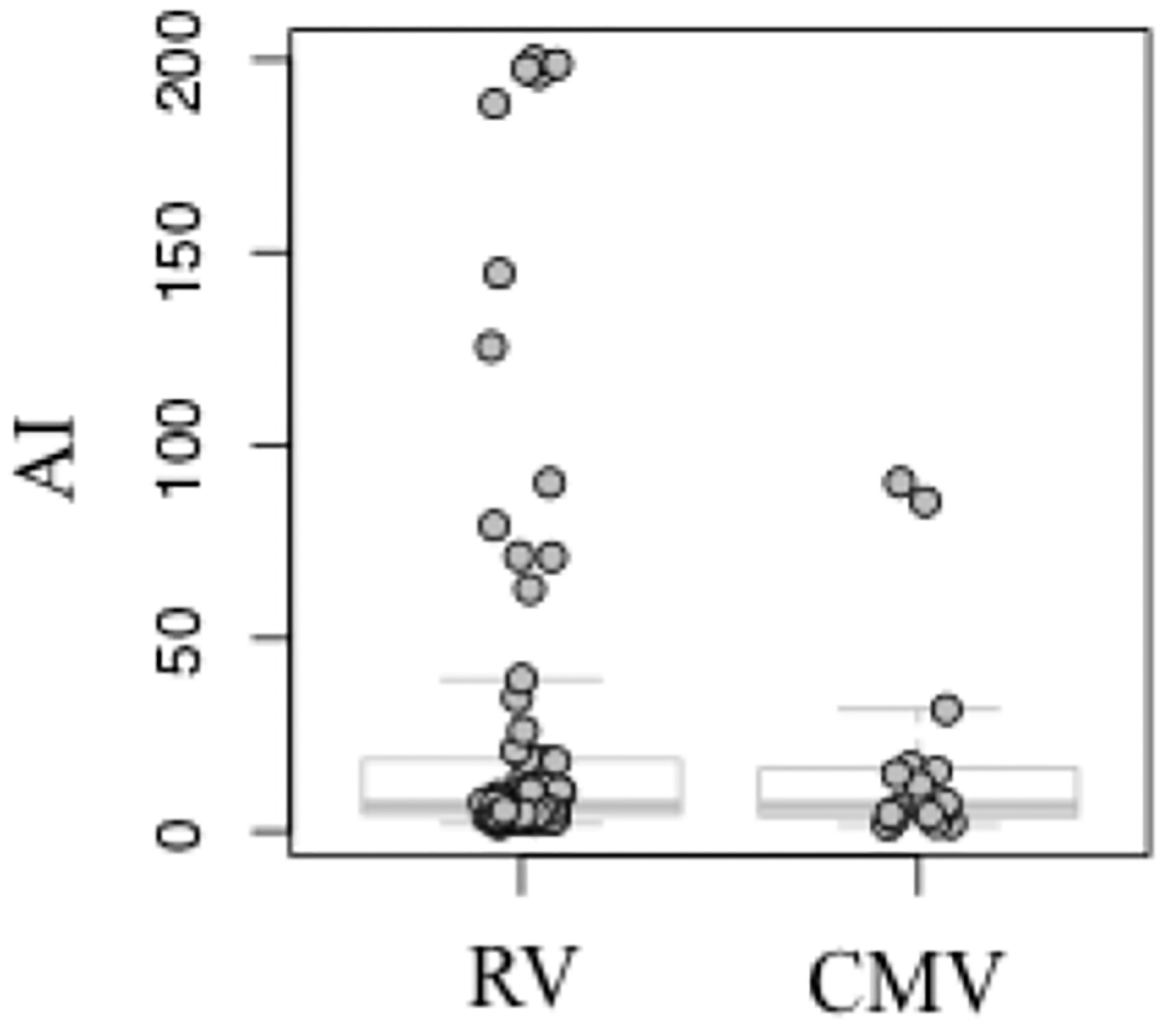
Antibody index (AI) of rubella (RV) and cytomegalovirus (CMV) in all 81 patients. AI was determined using the Goldmann-Witmer-Desmonts coefficient. The diagnosis for PSS was confirmed by AI > 3.0, ΔE >0.200 for CMV and for FU by AI >3.0, ΔE >0.200 for RV.

Antibodies against CMV were detected in aqueous humor of all 16 PSS patients, and RV antibodies were found in all 65 FU patients with the corresponding clinical findings (Fig 1). HSV and VZV antibodies were not detected in any patient. We defined the PSS group as CMV+ (CMV+ PSS) and the FU group as RV+ (RV+ FU). The PSS group consisted of 8 females and 8 male patients, while the FU group included 27 females and 38 male patients. Mean age was 48 years (range 21–87). Further baseline data are summarized in Table 1.

**Table 1:** Baseline demographics and clinical presentation of all patients.

	PSS (n = 16)	FU (n = 65)
Median age at time of sample collection (years)	47.5	47.0
Range	21 to 84	19 to 87
Male gender, no. (%)	8 (50)	38 (58)
A) 0.5 to +1 cells in anterior chamber^18^, no. (%)	11 (69)	22 (34)
B) Endothelial precipitates, no. (%)	12 (75)	50 (77)
C) Absence of posterior synechiae, no. (%)	13 (81)	63 (95)
D) Median of highest IOP (mmHg)	22	16
Range	11 to 50	9 to 58
E) Heterochromia and/or iris stromal atrophy, no. (%)	1 (6)	42 (65)
F) Cataracta subcapsularis, no (%)	2 (12)	28 (43)
Phakic, no. (%)	10 (63)	16 (25)
Pseudophakic, no. (%)	4 (25)	21 (32)
G) Anterior vitreous involvement, no (%)	0 (0)	33 (51)

Seven patients in the PSS group received oral acetazolamide, while another 6 were on topical anti-glaucoma treatment and 3 patients did not receive any IOP lowering medications prior to obtaining aqueous humor samples. Twelve patients received topical steroid drops. In the FU group four patients were treated with acetazolamide, six patients received topical anti-glaucoma medications, and 2 patients received topical steroids. As a result, IOP levels at day of surgery were decreased to normal in all patients, although they had been elevated in some patients beforehand.

A third group of cataract patients included 4 females and 7 males acted as controls. In this group 11 eyes (11 patients) were included. Patients had a mean age of 73 year (ages 45–90). No CMV or RV antibodies were recorded in this group.

The levels of immune mediators in the PSS and FU groups are summarized in Table 2.

**Table 2:** Summary of cytokine levels (pg/mL) in all measured aqueous humor, using nonparametric Mann-Whitney-Test.

Mediators	PSS	FU	Controls	p-value		
	Median (Range)	Median (Range)	Median (Range)	PSS vs. Controls	FU vs. Controls	PSS vs. FU
Eotaxin	746.9 (0–1427)	274.2 (0–1469)	1.9 (0–433)	0.0002	0.0222	0.0003
FGFbasic	8.1 (0–64)	0.0 (0–65)	0.0 (0–89)	0.7768	0.2447	0.3240
G-CSF	326.1 (0–9331)	108.2 (0–37569)	0.0 (0–983)	0.0164	0.0451	0.1121
GM-CSF	0.0 (0–154)	0 (0–309)	0.0 (0)	0.1225	0.5844	0.0719
IL-1β	0.85 (0–12)	0.0 (0–22)	0.0 (0–3)	0.4306	0.2637	0.6104
IL-1RA	73.4 (0–899)	4.3 (0–1002)	0.0 (0–25)	<0.0001	0.0073	0.0077
IL-2	0.0 (0–412)	0.8 (0–502)	0.0 (0)	0.0216	<0.0001	0.8913
IL-4	8.9 (0–63)	0.3 (0–94)	0.0 (0)	0.0040	0.0022	0.2390
IL-5	27.9 (0–53)	2.3 (0–130)	0.0 (0–2)	<0.0001	0.0003	0.0076
IL-6	548.5 (637–24295)	707.0 (7–25160)	149.5 (0–610)	0.0144	<0.0001	0.6495
IL-7	672.7 (430–1122)	737.2 (0–2712)	677.6 (356–1121)	0.9321	0.6699	0.5388
IL-8	199.4 (6–1337)	72.4 (0–1724)	56.5 (0–158)	0.0089	0.1441	0.0233
IL-9	27.3 (0–171)	0.6 (0–362)	0.0 (0–0.6)	0.0007	0.0034	0.2624
IL-10	33.4 (0–421)	1.6 (0–572)	1.6 (0–101)	0.3070	0.4138	0.0383
IL-12	243.5 (37–533)	196.7 (0–1069)	191.8 (74–573)	0.7160	0.5045	0.5947
IL-13	314.7 (0–1047)	271.3 (0–2425)	134.7 (80–373)	0.1182	0.0350	0.9649
IL-15	45.0 (3–196)	49.8 (0–318)	0.0 (0–49)	0.0003	<0.0001	0.8995
IL-17	0.0 (0–442)	0.0 (0–2207)	0.0 (0)	0.2479	0.4657	0.2375
INF-γ	174.9 (0–1106)	25.4 (0–1989)	0.0 (0–153)	0.0003	0.0009	0.0619
IP-10	126350 (0–934873)	57400 (2–886442)	5748 (1802–15169)	<0.0001	<0.0001	0.0036
MCP-1	1267 (320–4685)	1561 (8–5482)	957.6 (439–1643)	0.1623	0.0013	0.2043
MIP-1α	113.4 (0–731)	155.4 (0–1523)	0 (0–82)	0.0002	<0.0001	0.2715
MIP-1β	372.8 (126–1342)	286.2 (8–1451)	218 (69–465)	0.0565	0.0709	0.5588
PDGF	74.3 (0–632)	2.2 (0–715)	0.0 (0)	0.0019	<0.0001	0.5791
RANTES	44.2 (0–857)	65.7 (0–416)	0 (0–80)	0.0003	<0.0001	0.5033
TNF-α	25.1 (7–96)	27.2 (0–109)	0.0 (0–2)	<0.0001	<0.0001	0.6952
VEGF	1646 (89–5603)	1302 (0–27671)	1294 (0–1923)	0.4809	0.4390	0.8716

### T helper 1 (T_H_1) cytokines

**Fig 2:**
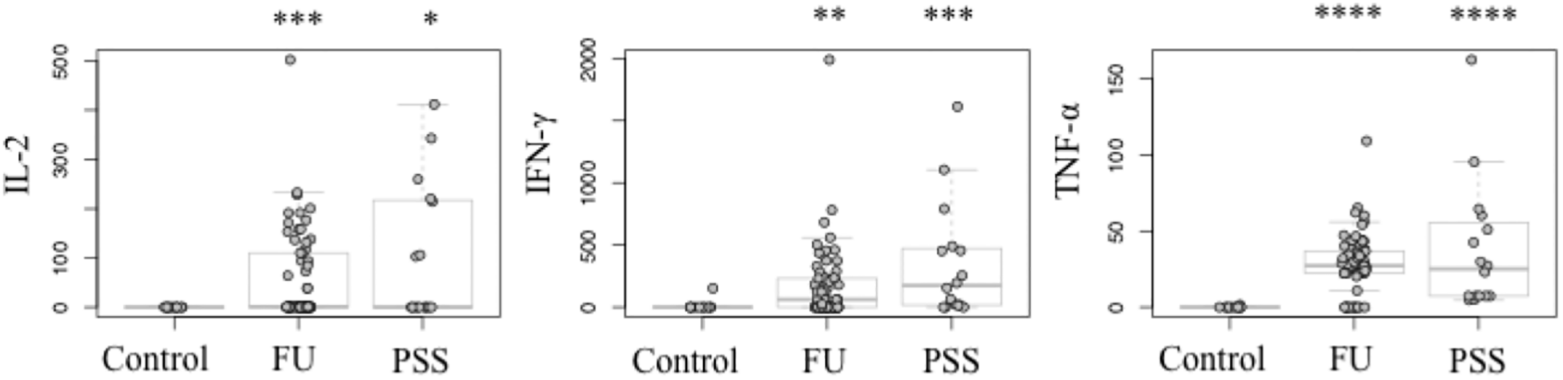
Increased mediator concentrations of T helper 1 cells for cytomegalovirus positive Posner-Schlossman-Syndrome (PSS) and rubella positive Fuchs´ Uveitis (FU) compared to the control group. The concentrations (pg/mL) were compared between PSS and FU vs. controls by nonparametric Mann-Whitney-Test. *P < 0.05, **P < 0.01, ***P < 0.001, ****P < 0.0001.

As shown in Fig 2, median concentrations of IL-2 (PSS: 0 [0–412] pg/mL; FU: 0.8 [0–502] pg/mL), IFN-γ (PSS: 174.9 [0–1106] pg/mL; FU: 25.4 [0–1989] pg/mL) and TNF-α (PSS: 25.1 [7–96] pg/mL; FU: 27.2 [0–109] pg/mL) were significantly higher in both groups compared to controls (IL-2: 0 [0] pg/mL; IFN-γ: 0.0 [0–153] pg/mL; TNF-α: 0 [0–2] pg/mL). IL-7 and IL-12 showed no apparent change. In all three groups, the range of median concentrations for IL-7 was 672–737 pg/mL and for IL-12 was 191–243 pg/mL (Table 2).

### T helper 2 (T_H_2) cytokines

The median concentrations of IL-4 (PSS: 8.9 [0–63] pg/mL; FU: 0.3 [0–94] pg/mL) and IL-5 (PSS: 27.9 [0–53] pg/mL; FU: 2.3 [0–130] pg/mL) were also significantly higher in both groups compared to controls (IL-4: 0 [0] pg/mL; IL-5: 0 [0–2] pg/mL), but IL-13 (271.3 [0–2425] pg/mL) was found to be increased only in FU (134.7 [80–373] pg/mL). Median concentrations of IL-10, also a member of the type 2 cytokine family, differed between PSS (33.4 [0–421] pg/mL) and FU (1.6 [0–572] pg/mL), but not between FU and controls (1.6 [0–101] pg/mL). Additionally, IL-5 median concentrations were significantly different between PSS (27.9 [0–53] pg/mL) and FU (2.3 [0–130] pg/mL) (Table 2).

### Anti- and pro-inflammatory mediators

**Fig 3:**
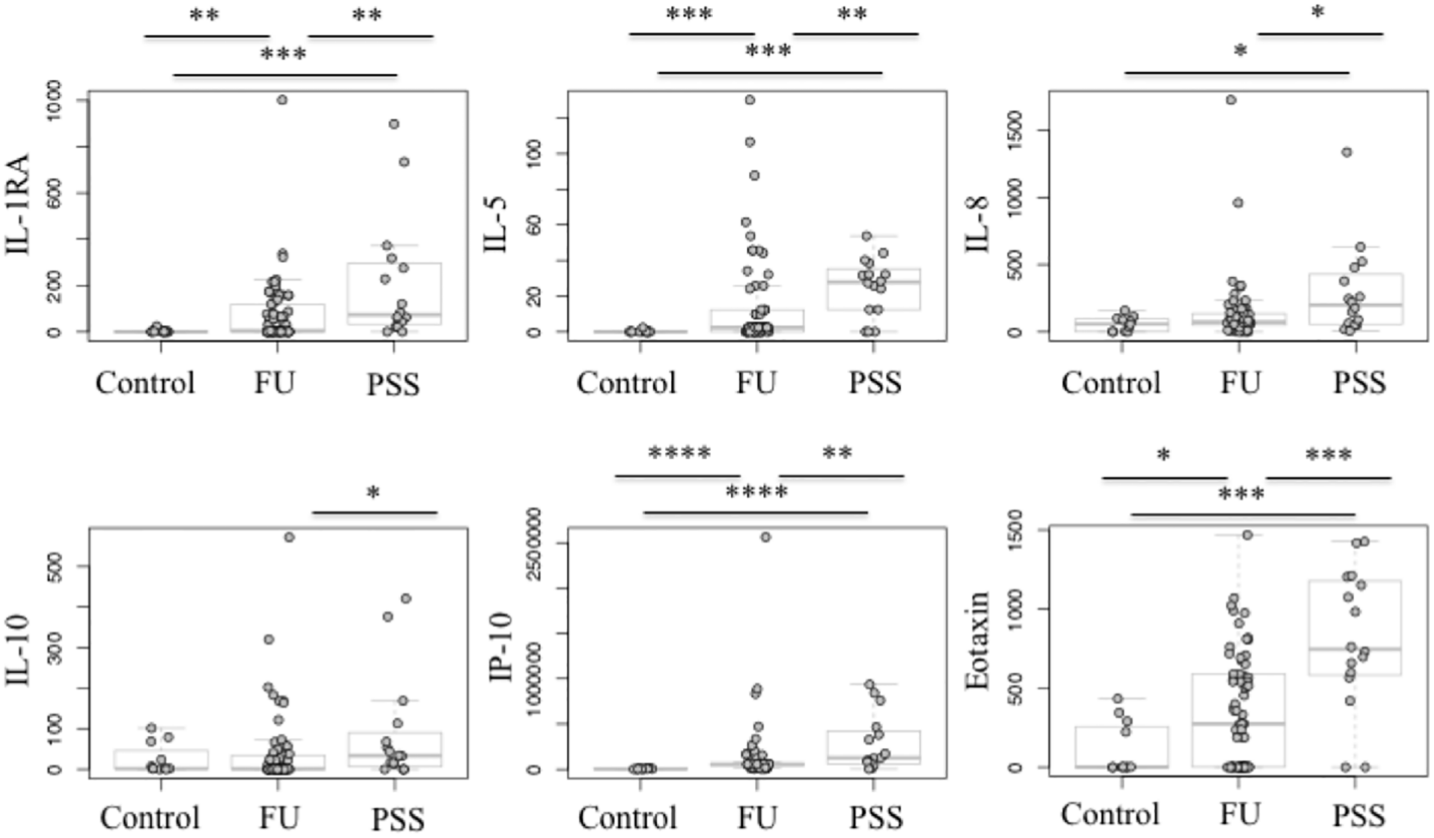
Mediators’ concentrations for cytomegalovirus positive Posner-Schlossman-Syndrome (PSS) and rubella positive Fuchs´ Uveitis (FU) and control group. The concentrations (pg/mL) were compared between PSS and FU vs. controls, and PSS vs. FU by nonparametric Mann-Whitney-Test. *P < 0.05, **P < 0.01, ***P < 0.001, ****P < 0.0001.

Both PSS and FU groups showed a significant increase in IL-1RA (PSS: IL-1RA: 73.4 [0–899] pg/mL; FU: 4.3 [0–1002] pg/mL), IL-6 (PSS: 548.5 [637–24295] pg/mL; FU: 707.0 [7–25160] pg/mL) and MIP-1α (CCL3) (PSS: 113.4 [0–731] pg/mL; FU: 155.4 [0–1523] pg/mL) median concentrations compared to controls (IL-1RA: 0 [0–25] pg/mL; IL-6: 149.5 [0–610]; MIP-1α: 0 [0–82] pg/mL) (Median [range]). IL-8 (CXCL8) was significantly increased in PSS (199.4 [6–1337] pg/mL) compared to controls (56.5 [0–158] pg/mL). Significant differences between PSS and FU were seen in IL-1RA (p = 0.0077) and IL-8 (CXCL8) (p = 0.0233) (Fig 3). Among all three groups differences in median concentration of IL-1β were not significant (Table 2).

### IL-15, IL-17, IL-9

In none of the groups, expression of IL-17 was significantly elevated (PSS: 0 [0–442] pg/mL; FU: 0.0 [0–2207] pg/mL; controls: 0 [0] pg/mL). But in both PSS and FU median concentrations of IL-9 (PSS: 27.3 [0–171] pg/mL; FU: 0.6 [0–362] pg/mL) were significantly increased in comparison to controls (0 [0–0.6] pg/mL). The same was found to be true for IL-15 (PSS: 45.0 [3–196] pg/mL; FU: 49.8 [0–318]; controls: 0.0 [0–49]) (Table 2 and S1 Fig).

### Chemokines

Eotaxin, known as (CCL11), showed a significant increase in PSS (746.9 [0–1427] pg/mL) compared to FU (274.2 [0–1469] pg/mL). Also, IP-10 (CXCL10) median concentrations were higher in PSS (126350 [0–934873] pg/mL) than in FU (57400 [2–886442] pg/mL) and controls (5748 [1802–15169] pg/mL). Another chemokine, MCP-1 showed significant differences between PSS (1267 [320–4685] pg/mL) and FU (1561 [8–5482] pg/mL), compared to controls (957.6 [439–1643] pg/mL). A significant difference in median concentrations of RANTES was also seen in PSS (44.2 [0–857] pg/mL) and FU (65.7 [0–416] pg/mL) compared to controls (0 [0–80] pg/mL) (Table 2).

### Growth factors

G-CSF showed higher levels in PSS (326.1 [0–9331] pg/mL) and FU (108.2 [0–37569] pg/mL), compared to controls (0 [0–983] pg/mL). Median concentrations of PDGF were also higher in both groups than in controls (PSS: 74.3 [0–632] pg/mL; FU: 2.2 [0–715] pg/mL; controls: 0 [0] pg/mL). FGFbasic, GM-CSF, and VEGF did not show significant between group differences (Table 2).

### Cluster analysis

**Fig 4:**
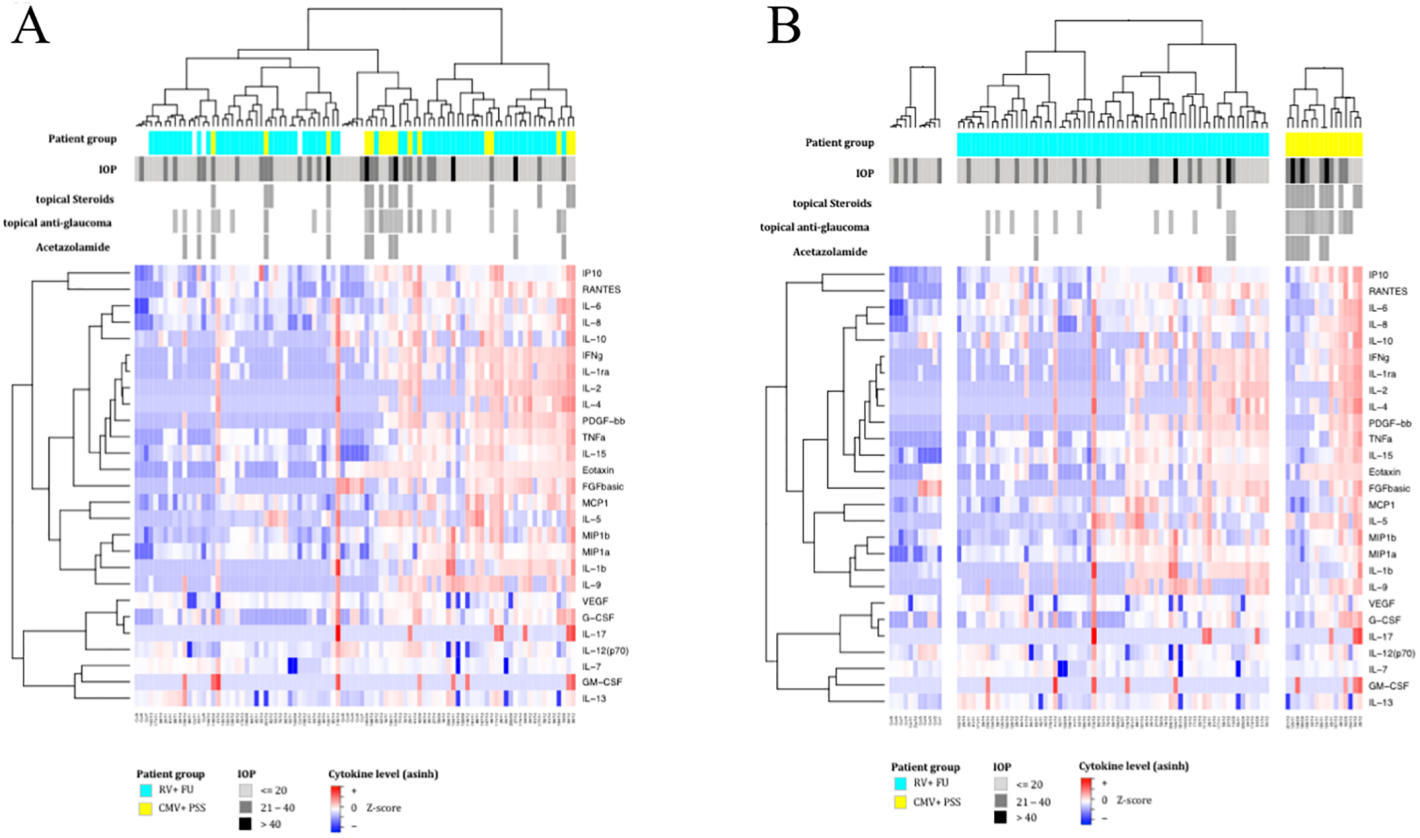
Cluster analysis of aqueous cytokine levels in Posner-Schlossman-Syndrome (PSS), Fuchs´Uveitis (FU) patients, and control group. The measured values of analyzed concentration are represented in heatmaps with colors according to (per-analyte) mean-centered and sigma-normalized data (Z-score) after asinh transformation of the raw values. The height of the dendrogram represents the dissimilarity between clusters of patient samples (columns) according to the profile of secreted immune mediators (rows). The top-bars indicate the PSS (yellow), the FU (cyan) and control group (white), as well as IOP level of each individual (light grey: IOP< = 20, grey: IOP>20, black: IOP>40), topical steroid treatment (white: no treatment), topical anti-glaucoma treatment (white: no, light grey: Brimonidine/Timolol, grey: Dorzolamide/Timolol), and systemic anti-glaucoma treatment with acetazolamide (white: no treatment). **A)** Simultaneous analysis of data from all three groups indicates that one part of PSS group with high IOP and low levels of mediators fall into a single cluster with a distinct immune mediator expression profile. **B)** Clustering performed in each group separately shows that within the PSS group patients with mostly low levels of mediators have also high IOP and have been treated with acetazolamide.

Heatmaps were created representing immune mediator levels in all individual samples acquired from patients with PSS or FU and healthy donors (Fig 4). Hierarchical clustering across all samples segregated patients into groups with similar cytokine profiles. Interestingly, half of patients with positive CMV-specific antibodies fell into the same cluster indicating a high dissimilarity with regard to their aqueous immune mediator composition (Fig 4A). Such patients also had high IOP levels. When performing clustering within each patient group individually (Fig 4B), PSS patients also segregated into two clusters with mostly low or high cytokines levels, which in this group corresponded to either high IOP and acetazolamide treatment or low IOP and absence of acetazolamide, respectively.

### Correlation with IOP

There was a significant correlation only between AI and TNF-α in FU patients. We did not observe any correlation of AI and cytokines in PSS patients. Furthermore, there was no correlation between the SUN score for anterior chamber inflammation and certain cytokines.

**Fig 5:**
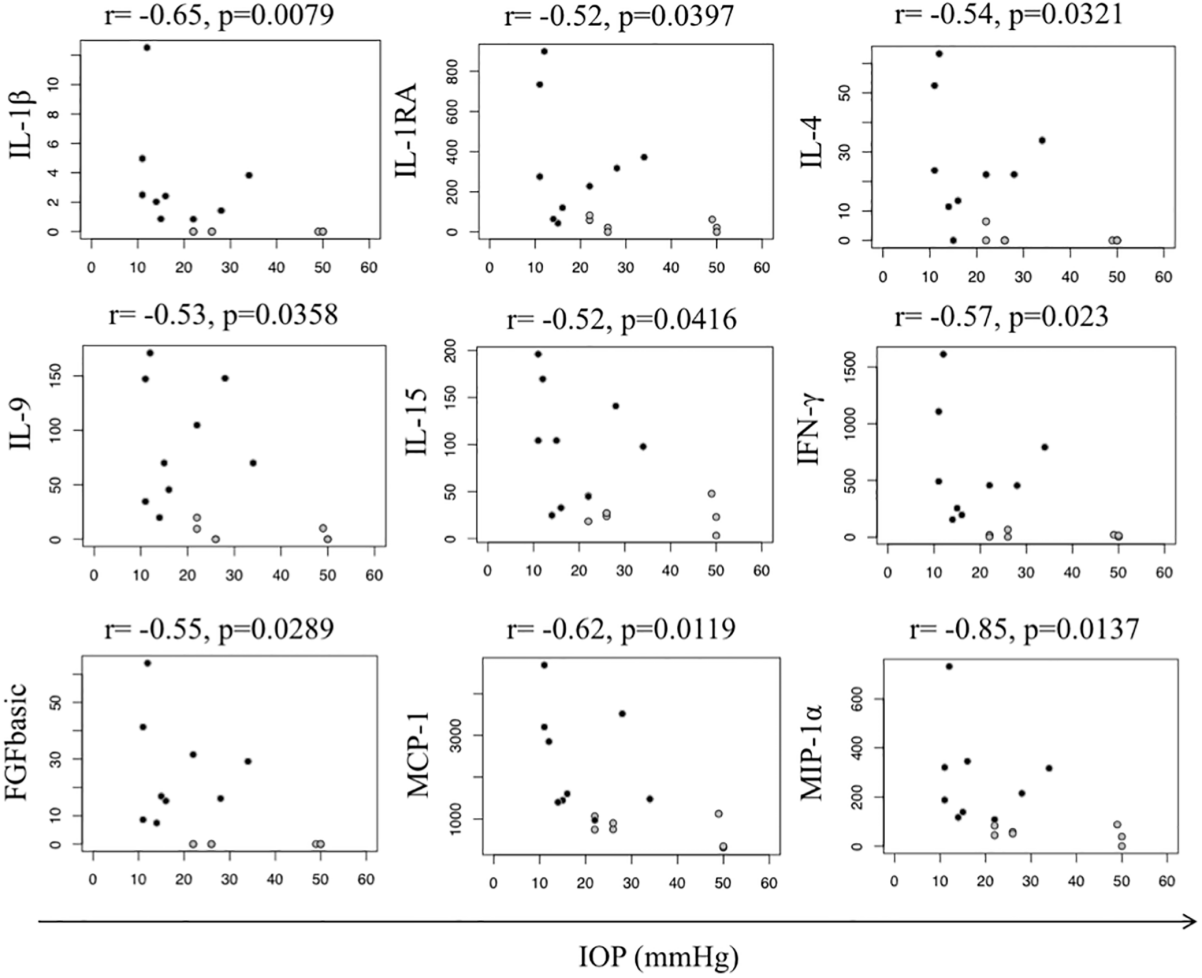
Spearman correlations of median concentrations mediators (pg/mL) with intraocular pressure (IOP) in the Posner-Schlossman-Syndrome (PSS) group. Immune mediators (IL-b, IL-1RA, IL-4, IL-9, IL-15, IFN-γ, FGFbasic, MCP-1 and MIP-1α) correlate negatively with IOP in the total PSS group (black and light grey). Patients treated with acetazolamide (light grey) show lower immune mediators levels. mmHg = millimeter of mercury.

In the PSS group, IL-1β (r = -0.65, p = 0.0079), IL-1RA (r = -0.52, p = 0.0397), IL-4 (r = -0,54, p = 0.0321), IL-9 (r = -0.53, p = 0.0358), IL-15 (r = -0.51, p = 0.0435), FGFbasic (r = -0.55, p = 0.0289), IFN-γ (r = 0.57, p = 0.023), MCP-1 (r = -0.62, p = 0.0119), and MIP-1α (r = -0.85, p = 0.0137) were found to be negatively correlated with IOP values (Fig 5). The 3 PSS “naïve” study patients not receiving glaucoma treatment did not negatively confound the correlation, since they presented with the lowest IOP values. However, as also indicated in the heatmap, PSS patients receiving acetazolamide showed significantly lower cytokine concentrations.

## Discussion

Posner-Schlossman-Syndrome and Fuchs´Uveitis reveal frequent similarities which lead to a difficult identification of the actual etiology. Clinically, both disorders have a predominantly unilateral manifestation in common, presenting with recurrent flare ups and only mild intraocular inflammation. A striking difference between PSS and FU is the glaucomatocyclitic crisis, an acute onset of IOP elevation in PSS. Compared to this, FU patients are markedly characterized by a long-standing, low-grade inflammation, and less acute elevated IOPs. This prolonged inflammation and persistently higher IOP more often result in chronic glaucoma in FU patients [2].

These clinical findings raise the question, which role CMV and RV play in this context, and whether CMV and RV are a “bystander” or activator of inflammation in the anterior chamber. In previous studies, immune mediators in aqueous humor of different uveitis entities were reported, but so far, no differentiation between these infections were proven [10–12,17,18].

In our study, we detected significantly increased levels of almost all immune mediators in PSS and FU compared to controls. Several immune mediators were expressed distinctly differently in PSS compared to FU. In general, the inflammation was promoted by a strong T_H_1 response in both disorders (Fig 2). Particularly, significant higher IFN-γ levels were measured in PSS. As an antiviral cytokine by inhibiting viral replication, IFN-γ was also reported in patients with ocular toxoplasmosis, intermediate uveitis, and viral uveitis [19,20]. Although T_H_2 response was significantly lower than T_H_1, we observed an upregulation of IL-4 and IL-5 in PSS and FU for down-regulating the local inflammatory process of T_H_1. Interestingly, IL-5 and IL-10 levels were also significantly higher in PSS compared to FU. IL-5 seems to act not only as a helper factor, serving to sustain B-cell proliferation, but also possesses antiapoptotic effects [21]. Lahmar et al. reported high levels of IL-5 in human ocular toxoplasmosis [19]. High IL-10 levels are mainly associated with active infectious uveitis and considered to be important in early stage of infection [10,22]. We assume that high levels of IFN-γ, IL-5, and IL-10 in PSS imply a distinctively acute inflammation in PSS triggered by CMV. Although FU revealed elevated cytokine levels, but not as clearly as PSS, so that we conclude RV triggers a chronic, persistent inflammation. Our assumption is also based on other results in our study. The levels of proinflammatory IL-1RA, IL-6, IL-8 (CXCL8), and MIP-1α (CCL3) were significantly higher in PSS (Table 2) so that CMV might act as an activator of these cytokines. According to the heatmap, IL-6 and IL-8 are expressed simultaneously. We suggest that IL-6, IL-8, and MIP-1α control the migration and infiltration of monocytes/macrophages during inflammation and contribute to the viral response in PSS. High IL-6 levels induce an increase in intraocular inflammation, as seen in idiopathic uveitis and ocular infection such as toxoplasmosis gondii [11,13,23]. Moreover, we assume that CMV induces IL-8 and IL-1RA production. Murayama and colleagues reported similar data suggesting that CMV infection causes IL-8 production in human monocytic cell lines and increases gene expression of receptor of IL-8 in fibroblast cell lines [24]. IL-1RA is secreted by a variety of cells to directly interfere with the pro-inflammatory IL-1 cytokine. It is highly expressed in patients with sepsis, chronic rheumatic disease, and graft-versus-host disease patients with CMV primary infection or CMV reactivation [24, 25]. Zaho et al. did report increased IL-1RA levels in the aqueous humor of patients with HLA-B27-associated autoimmune uveitis. This is interesting to note, as it may indicate anti-inflammatory response is initiated during active uveitis, in order to suppress local response and protect against inflammation [26]. Our results also show an increase in IL-1RA levels especially in the PSS group (Fig 3). Thus, IL-1RA and IL-8 may very well act as markers in aqueous humor for acute exacerbation and inflammation in PSS. Overall, half of patients with PSS showed a similar within group composition of immune mediators in cluster analysis (Fig 4A). Most of them also suffered from high IOP. Our heatmap substantiated the relationship between immune mediators and IOP in the PSS group. We observed a negative correlation between mediators and IOP in the PSS group. A trabecular meshwork (TM) obstruction could be the reason for the increased IOPs. The disruption of the blood aqueous barrier allows entry of inflammatory cells in the aqueous humor and a variety of mediators are released by resident cells of the ciliary body, which may lead to swelling of trabecular lamellae, increase the permeability of endothelial cells with subsequent edema formation in surrounding structures. This results in a decreased outflow of aqueous humor and leads to the very high IOP increases [27,28]. Probably, due to a reduction of the aqueous humor drainage from the anterior chamber and degradation of mediators or rather apoptosis, we could have observed a negative correlation between concentrations of mediators and increased IOP. Even more interestingly, we observed significantly lower mediator levels in patients treated with acetazolamide (Figs 4A + 4B and 5). So far it has not been reported that acetazolamide has any impact on the immune mediators in aqueous humor. Only one study described that acetazolamide decreased proinflammatory cytokines, such as MCP-1, IL-1 β, TNF- α, and IFN- γ in rat lungs which presumably protected hypoxia rats. The reduced cytokine levels in rat lungs after acetazolamide treatment is discussed by the authors as another possible explanation for effectiveness of acetazolamide in acute mountain sickness [29]. In general, it is known that acetazolamide decreases the production of aqueous humor, thus reducing IOP. But the reletionship of acetazolamide and lowering of immune mediators in aqueous humor has not yet been investigated.

Similar to all previous reports, our results must be interpreted carefully. Data used are in fact just a snap shot of immune mediator levels at a single time point. The study has also several limitations. Even though our patient cohort is the largest so far, sample sizes and exploratory analysis were still limited. Therefore, findings will have to be validated in a larger cohort of patients.

### Conclusion

There are remarkable similarities between clinical presentation and cytokine profiles in PSS and FU. Still there are also some significant differences. Both PSS and FU were characterized by T_H_1 cells mediated immune response, but there seems to be a stronger cytokine response in PSS. Moreover, increased concentrations of IL-1RA, IL-8, IL-10, and IP-10 indicate a more active inflammation in PSS (with CMV as an activator). In contrast, FU presents as a chronic persistent inflammation (with RV as a bystander). Additionally, acetazolamide appears to have an influence on immune mediators in aqueous humor. Further investigations are needed to confirm these new findings.

## Supporting information

S1 Fig Mediators`concentrations for cytomegalovirus positive Posner-Schlossman-Syndrome (PSS) and rubella positive Fuchs´ Uveitis (FU) and control group. The concentrations (pg/mL) were compared between PSS and FU vs. controls, and PSS vs. FU by nonparametric Mann-Whitney-Test. *P < 0.05, **P < 0.01, ***P < 0.001, ****P < 0.0001.
(TIFF)

S1 Dataset Minimal dataset. (XLSX)
