# Supplementary figures and images for: Different composition of intraocular immune mediators in Posner-Schlossman-Syndrome and Fuchs’ Uveitis
Source: PLoS One. 2018 Jun 26;13(6):e0199301. doi: 10.1371/journal.pone.0199301 (PMC6019249; doi:10.1371/journal.pone.0199301)

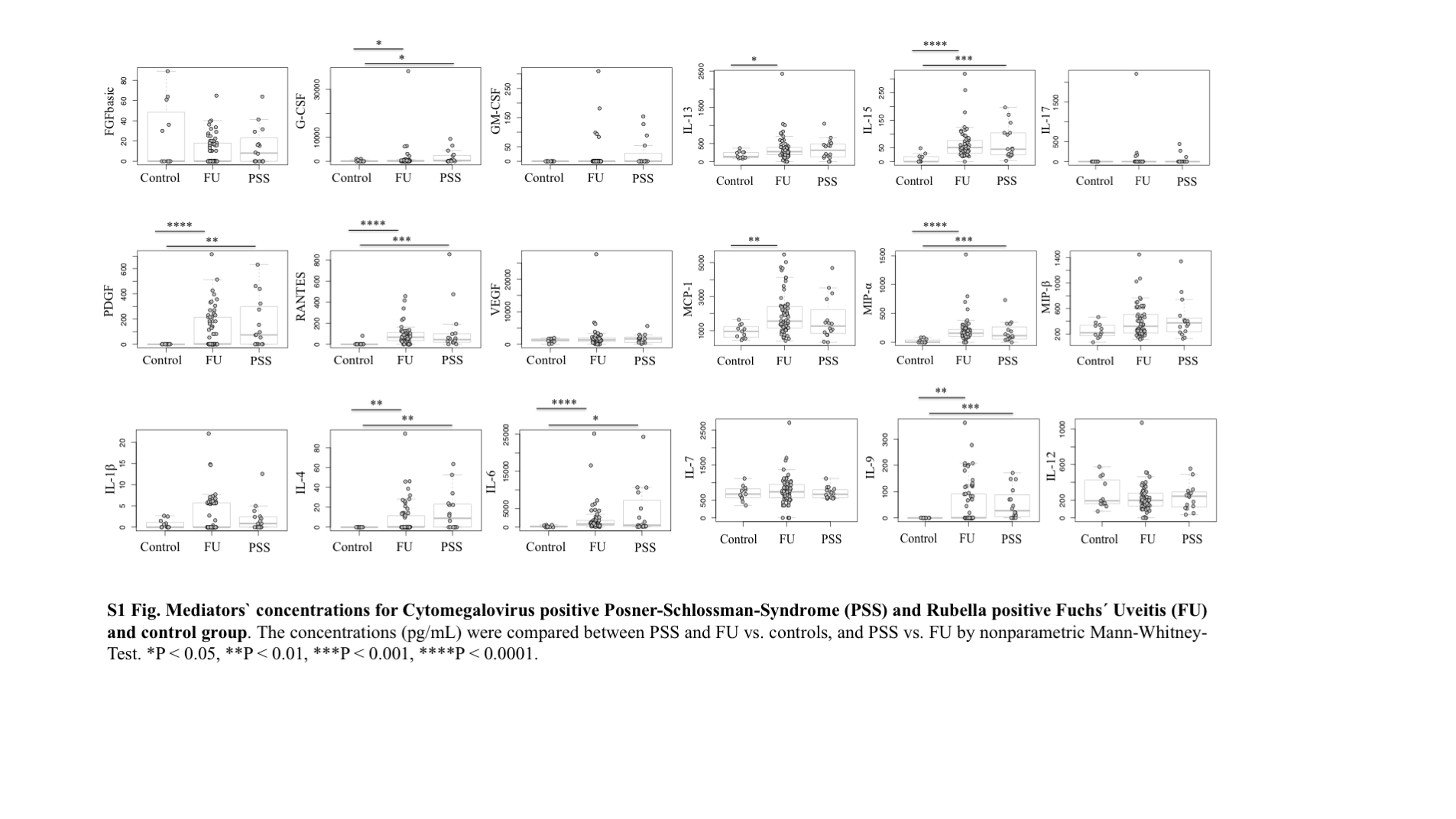

Supplement: S1 Fig — The concentrations (pg/mL) were compared between PSS and FU vs. controls, and PSS vs. FU by nonparametric Mann-Whitney-Test. *P < 0.05, **P < 0.01, ***P < 0.001, ****P < 0.0001. (TIFF) [file pone.0199301.s001.tiff]
